# Non-Neutral Vegetation Dynamics

**DOI:** 10.1371/journal.pone.0000078

**Published:** 2006-12-20

**Authors:** Marco Marani, Tommaso Zillio, Enrica Belluco, Sonia Silvestri, Amos Maritan

**Affiliations:** 1 Dipartimento di Ingegneria Idraulica, Marittima, Ambientale e Geotecnica (IMAGE), and International Center for Hydrology, Università di Padova Padova, Italy; 2 International School for Advanced Studies (SISSA) Trieste, Italy; 3 Servizio Informativo - Consorzio Venezia Nuova (CVN), Magistrato alle Acque di Venezia Venezia, Italy; 4 Coordinamento Nazionale Insegnanti Specializzati (CNIS), Unita' di Padova Padova, Italy; University of Sheffield, United Kingdom

## Abstract

The neutral theory of biodiversity constitutes a reference null hypothesis for the interpretation of ecosystem dynamics and produces relatively simple analytical descriptions of basic system properties, which can be easily compared to observations. On the contrary, investigations in non-neutral dynamics have in the past been limited by the complexity arising from heterogeneous demographic behaviours and by the relative paucity of detailed observations of the spatial distribution of species diversity (beta-diversity): These circumstances prevented the development and testing of explicit non-neutral mathematical descriptions linking competitive strategies and observable ecosystem properties. Here we introduce an exact non-neutral model of vegetation dynamics, based on cloning and seed dispersal, which yields closed-form characterizations of beta-diversity. The predictions of the non-neutral model are validated using new high-resolution remote-sensing observations of salt-marsh vegetation in the Venice Lagoon (Italy). Model expressions of beta-diversity show a remarkable agreement with observed distributions within the wide observational range of scales explored (5⋅10^−1^ m÷10^3^ m). We also consider a neutral version of the model and find its predictions to be in agreement with the more limited characterization of beta-diversity typical of the neutral theory (based on the likelihood that two sites be conspecific or heterospecific, irrespective of the species). However, such an agreement proves to be misleading as the recruitment rates by propagules and by seed dispersal assumed by the neutral model do not reflect known species characteristics and correspond to averages of those obtained under the more general non-neutral hypothesis. We conclude that non-neutral beta-diversity characterizations are required to describe ecosystem dynamics in the presence of species-dependent properties and to successfully relate the observed patterns to the underlying processes.

## Introduction

Beta-diversity, the spatial structure of species diversity, is a key to the understanding of competitive strategies of organisms in complex ecosystems [1]. The clustering of conspecific individuals, for example, bears the signatures of the processes regulating recruitment modes and rates, and the spatial range of their effectiveness (e.g. of seed dispersal vs. vegetative regeneration in plants). However, such signatures do not have a straightforward interpretation and require a deeper quantitative understanding in terms of the strategies of competing species. The interpretation of observed patterns is often based on the neutral theory of biodiversity [2], which provides useful descriptions of ecosystem dynamics, e.g. in the case of tropical forests and coral reefs [3]–[6]. However, neutral models cannot, by construction, account for differences in habitat properties, plant physiologies or competitive abilities, and their applicability has been recently questioned [7]. Observational characterizations of the spatial biodiversity have also been recently achieved for microbial communities [8] and savanna vegetation [9], [10] providing new evidence about the spatial ecological structure in these environments. However, a comprehensive theory of the spatial organization of biodiversity linking observed patterns to the processes generating them is still lacking and requires the development of new models able to realistically represent heterogeneous competitive strategies.

Here we introduce a new non-neutral model of plant competition, which accounts for differences in the species demographic characteristics, yet allowing the derivation of analytical solutions. The model is constructed to reflect the main characters of wetland vegetation, observations of which are used for a thorough quantitative validation. However, the model also provides indications of a general nature about the linkages between competitive abilities and the observed beta-diversity in ecological systems driven by cloning, seed dispersal and density-independent mortality.

## Materials and Methods

### Intertidal vegetation: Observed beta-diversity

**Figure 1:**
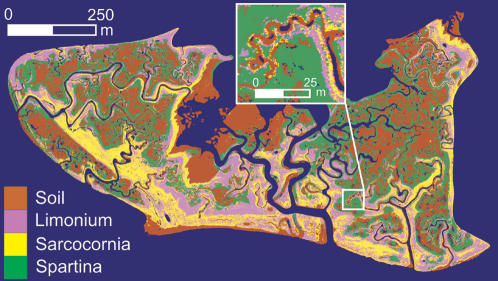
Vegetation map of the San Felice salt marsh in the Venice Lagoon (geometric resolution is 0.5 m, channels and creeks are in blue). The inset provides an indication of the high level of detail characterizing the data, which resolve small-scale channel and vegetation structures.

Coastal marshes host an extremely high biodiversity (often including rare or endangered species), exhibit one of the highest rates of primary production in the world and are experiencing a global decline [11], [12]. Furthermore, the explanation of the peculiar distribution of marsh species [13]–[15] may have broad implications for the search of the general neutral or non-neutral mechanisms leading to the observed spatial distribution of species diversity in ecosystems [16], [17]. We analyze here a map of the spatial distribution of vegetation species obtained from high-resolution remote sensing observations acquired in the Venice Lagoon [13]–[15], [18]. The map (Figure 1) was obtained by use of the Spectral Angle Mapper classifier [19] from multispectral data collected in October 2002. The difficult accessibility of intertidal zones and the interannual variability of species presence prevent accurate traditional field census studies, which require long periods of time to be completed in vast areas and thus do not yield an istantaneous characterization of the species spatial distribution. The accuracy achieved by recent remotely-sensed vegetation maps overcomes such sampling limitations, and allows the quantitative determination of the spatial distribution of the species ([18], also see Methods and Materials S1 for details on how the classification of remotely-sensed data was performed). Figure 1 shows that the halophytic species *Limonium narbonense*, *Sarcocornia fruticosa*, and *Spartina maritima* exhibit the typical patchy distribution associated to the *zonation* phenomenon [14]. Such patterns are the result of habitat characteristics and competition and are here analyzed within the 0.5 m–1 km range of scales. The accuracy of the vegetation map was verified by comparing the results of the classification with an ancillary dataset consisting of more than 17,900 reference 0.5 m×0.5 m pixels, in which the vegetation type was determined by field surveys. The comparison shows that the Spectral Angle Mapper classifier correctly identifies more than 86 % of the reference pixels, indicating that remotely sensed maps of intertidal vegetation indeed constitute a sound basis for statistical analyses.

**Figure 2:**
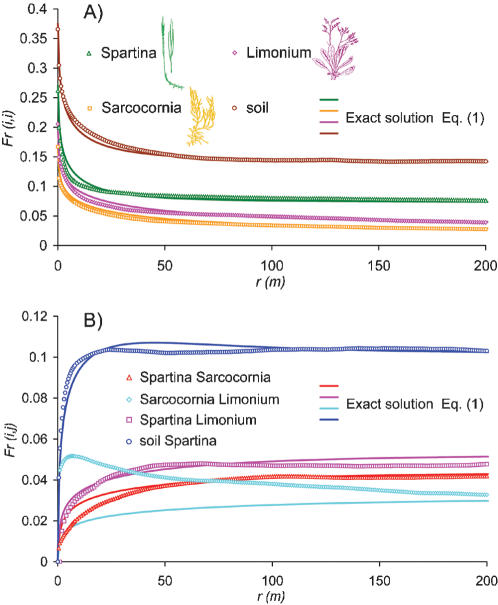
Beta-diversity of intertidal vegetation species. The solid symbols represent the probability of occurrence of species *i* and *j* at two sites separated by a distance *r* as computed from the vegetation distributions of Figure 1. The solid lines are plots of eqs. (1) from the model fitted on observations. Panel a) refers to the case in which *i = j*, (conspecific sites). In panel b), referring to the case *i≠j*, only one beta-diversity curve involving soil was indicated for clarity, the remaining ones being similar.

A characterization of the spatial distribution of species diversity is provided by the two-point correlation function given by the probability, *F_r_(i,j)*, that two sites separated by a distance *r* host species *i* and *j*. *F_r_(i,j)* functions were estimated from our observational data (Figure 2, hollow symbols) using a discrete step *Δr = 0.5 m* (the pixel size of the vegetation map), by sequentially considering each site in the map hosting species *i* (*j*), by computing the fraction of sites within the range *[r−Δr/2, r+Δr/2]* hosting species *j* (*i*), and by finally averaging over the entire domain. The value of *F_r = 0 _(i,i)* is equal to the average density of species *i* in the domain, whereas *F_r = 0_ (i,j) = 0* if *i≠j*. Furthermore, when *r* is large enough for species occurrence to be uncorrelated, *F_r_(i,j)* approaches the value *F_r = 0_ (i,i)·F_r = 0_ (j,j)*. The specific shape of *F_r_ (i,i)* depends both on how fast species presence becomes spatially uncorrelated and on the average density of each species. The presence of a local maximum in the *Sarcocornia*-*Limonium* beta-diversity (Figure 2) is indicative of a ‘preferential distance’ between the two species, which often occur in roughly parallel banded structures (see Figure 1), possibly due to the influence of topography on vegetation occurrence [14]. These analyses show that significant information on the spatial arrangement of species may be extracted from beta-diversity curves, but it is far from obvious how the competitive behaviour of different species may be inferred from them. It will later be seen that the development of a mathematical model for the collective behaviour of the ecosystem indeed provides a quantitative link between beta-diversity and the reproductive and competitive abilities of the different species. The wide differences in the beta-diversity curves in Figure 1, associated to similarly different spatial patterns, are the signature of fundamental differences in competitive strategies among the vegetation species. We suggest that the neutral theory of biodiversity cannot account for the observed differences in the spatial arrangement of wetland species, which thus require a non-neutral description. The development of a non-neutral model and the comparison of its predictions with those from a neutral one will be seen in the following to support this suggestion.

### The model

The stochastic model is defined on a regular lattice. Each site of the lattice represents a collection of plants in an ‘elementary’ area (e.g. of size 0.5 m×0.5 m, as in the remotely-sensed maps), and is characterized by its state *i*, representing bare soil (*i = 0*) and *S* vegetation species (*i = 1, 2, …, S*). Starting with an arbitrary initial species distribution (which have no influence on the stationary-state properties of the system), the model dynamics are defined by the following rules. At each time step the state of just one site, chosen at random, is updated. With probability *k* the state of the site is changed in the following manner: a) if the site is occupied by species *i*, it becomes empty with probability *d_i_* (and thus remains unchanged with probability *1−d_i_*); b) if the site is empty, it is colonized by species *i* with probability *v_i_* and remains empty with probability *1−Σ_i_v_i_*. The parameters *v_i_* represent the births from seed banks or long-range seed or rhizome fragment dispersal, quite effective in intertidal areas due to hydrodynamic transport [12], [20]. With probability *1−k* the site is colonized by vegetative growth and acquires the state of one of its nearest neighbours chosen at random. In principle, reproduction and death rates should be dependent on position, as they are influenced by the local environmental conditions. Indeed, intertidal vegetation species are known to occur within preferred soil elevation intervals [14], mainly as a result of the variable soil aeration determined by nontrivial feedbacks between halophytes and hydrologic fluxes [12], [21]–[23]. However, the species in Figure 1 are characterized by largely overlapping preferential soil elevation intervals, justifying the simplifying assumption of spatially-constant parameters (see Methods and Materials S1 for details). Furthermore, the possible time dependence of the model parameters has been neglected, postulating relatively steady habitat characteristics, supported by the relative stability of the observed overall vegetation spatial patterns on the annual scale and by a rapid adjustment of species presence to climatic or external perturbations. The observed beta-diversity, and the corresponding probability distributions *F_r_(i,j)* which will be later derived from the model, are therefore a synthesis of plant death/reproduction rates as jointly determined by species physiology and habitat characteristics within the specific site considered.

The model just introduced can be formulated in terms of a master equation (i.e. an equation describing the time evolution of species probabilities of occurrence [24], see Methods and Materials S1 for a detailed derivation), which is linear and amenable to analytical manipulations to obtain an explicit form of the stationary-state beta-diversity *F_r_(i,j)*. In particular, *F_r_(i,j)* can be expressed as the following linear combination of basis functions:1
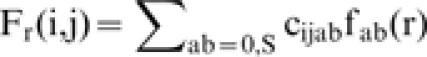



The basis functions are given by:2
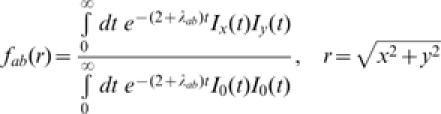
where *I_n_(t)* is the modified Bessel function of order *n* [25], and *λ_ab_* is the eigenvalue of the transition probability matrix associated to the master equation. The detailed expressions of the coefficients c_ijab_ are provided in Methods and Materials S1. Notice that the expression in eq. (2) is not isotropic because of the anisotropic structure of the lattice.

This formal problem can be easily overcome by the introduction of the angular average, *f(r, λ_a,b_)*, of the function *f(* ***r*** *, λ_a,b_)* for a fixed value *r = |* ***r*** *|*. However, *f(* ***r*** *, λ_a,b_)* is numerically found to be isotropic when *r* is greater than a few times the lattice cell size.

Because *F_r_(i,j) = F_r_(j,i)*, the number of independent curves that the model generates is *(S+2)(S+1)/2*. By suitably rescaling the parameters of the master equation, it is seen that the model behavior depends on only two parameters per species: *v′_i_ = kv_i_/(1−k)* and *d′_i_ = kd_i_/(1−k)*. The first parameter, *v′_i_*, expresses the ratio of the probability of birth (*kv_i_*) from dispersed seeds or plant fragments (‘non-local’ mechanism) to the probability (*1−k*) of propagule colonization (‘local’ mechanism). The second parameter, *d′_i_*, is the ratio between the probability (*kd_i_*) of plant death to the probability of ‘local’ propagule reproduction.

The model allows the explicit computation of the expected density for each species (see Methods and Materials S1 for details). If *a_i_ = v_i_/d_i_*, one finds that the expected density of species *i* is:3
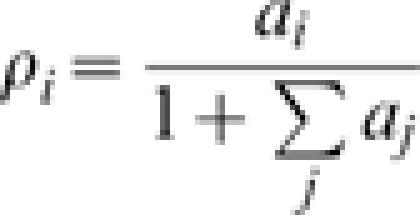
while the expected density of bare soil is 
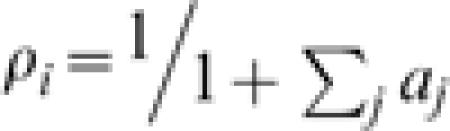
. We can thus derive expressions for the relative sensitivity of the density of species *i* to a variation of the parameter *a_k_* of the generic species *k*, defined as: 
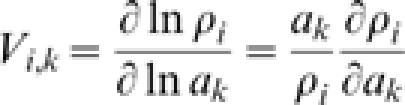
. When *k = i*, it is V*_i,i_* = 1−ρ*_i_*, showing that more abundant species are less sensitive to variations in their own birth/death ratios. When *k≠i* it is *V_i,k_* = −ρ*_k_*, showing that the sensitivity of the density of any species *i* to variations in the birth/death ratio of another species, *k*, is greater when the latter is more abundant. It may also be seen that such sensitivity is independent of the species *i* considered, and that, due to the negative sign in the expression of *V_i,k_*, an increase in the birth/death ratio of a species always determines a decrease in the abundance of the remaining species as a result of competition.

In order to explore the ability of a neutral model to describe the observed beta-diversity, we will also apply the model introduced above under the hypothesis that all species be characterized by the same effective competitive strategies. This is obtained simply by imposing that the parameters for all the species be equal, i.e. *v′_i_ = v′* and *d′_i_ = d′*, 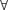 *i*. Comparisons between observations and predictions by the neutral and non-neutral versions of the model will be discussed below.

## Results and Discussion

The non-neutral model was fitted to the observations by minimizing the mean squared deviations between modelled (eq. 1) and observed *F_r_(i,j)* curves. Notice that the model has relatively few degrees of freedom (*2S* parameters, where *S = 3* in the present case) to fit the *(S+2)(S+1)/2* observed beta-diversity curves, and is thus quite parameter-parsimonious, dissipating doubts about a possible overfitting. Comparisons of modelled beta-diversity curves (Figure 2, solid lines) with observations (Figure 2, hollow symbols) show a remarkably good agreement over the entire range of scales available, which is quite indicative of the model descriptive capabilities if one considers the relatively small number of parameters involved. The only notable departure of the model from the observations regards the *Sarcocornia-Limonium* beta-diversity curve. The model does not reproduce the observed local maximum, suggesting that the latter (and the parallel banded structures to which it is associated) be induced by environmental controls not accounted for (by construction) in the model. The model allows further inferences about the competitive abilities of the different vegetation species. The values of the *v′_i_* parameters obtained by calibration (see Methods and Materials S1 for a list of all parameter values) were *4.51 ·10^−6^* for *Limonium narbonense*, *3.63 ·10^−6^* for *Sarcocornia fruticosa* and *1.26 ·10^−4^* for *Spartina maritima*. These small values indicate, under the stationary conditions considered, an evident general dominance of the (local) clonal vs. (non-local) seed/rhizome fragment dispersal reproduction strategies. This circumstance is consistent with broad halophyte physiological characteristics from field and laboratory observations [12], [20], [26]. However, it should be considered that our results provide a fully quantitative characterization, also accounting for factors which are otherwise difficult to evaluate, such as the rate of seedling survival, the efficiency of seed or vegetative material transport, etc. The *v′_i_* values indicate a relatively greater dominance of the local vegetative growth mechanism in the case of *Sarcocornia fruticosa* and *Limonium narbonense* as compared to *Spartina maritima*. Such an inference would hardly be possible by simple inspection or analysis of the beta-diversity curves. The model proposed is thus valuable in separating the effects of the competitive abilities of each species, which are otherwise intricately intermingled in the *F_r_(i,j)* functions of Figure 2, and in identifying and interpreting the signatures of local and non-local reproduction mechanisms in plant aggregation patterns.

Another interesting indication provided by the model stems from the similar values of the parameters *v′_i_* (ratios between the rates of local and non-local birth mechanisms) and *d′_i_* (ratios of death to local birth rates) for *Sarcocornia fruticosa* and *Limonium narbonense*. Such information, hardly obtainable from field experiments, indicates a close functional similarity of the two species and is useful when a distinction of functional types, rather than of species, is of interest.

The model we have proposed incorporates environmental spatial heterogeneities in the effective birth (from seeds/rhizome fragments and from propagules) and death rates, which summarize the species competitive abilities within the specific environment considered. The application of the model even to neighbouring marshes may thus lead to different parameter values for the same species, e.g. due to a different efficiency of the seed transport mechanisms, to varying seedling survival rates or to different rates of propagule production and establishment on different topographies (corresponding to different soil aeration conditions). This is an advantage of our approach, which allows the direct evaluation of the effectiveness of species competitive abilities in the actual environment, rather than in idealized laboratory conditions or within a limited and controlled study site.

**Figure 3:**
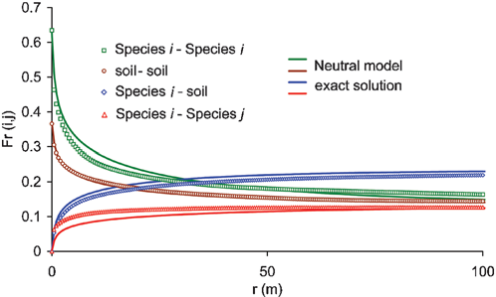
Beta-diversity of intertidal vegetation species under the neutral assumption. The hollow symbols represent observational values, while solid lines are plots of eq. (1) from the neutral model fitted on observations.

In spite of the functional similarity between *Sarcocornia fruticosa* and *Limonium narbonense* the differences with *Spartina maritima* are major, in terms of average density, beta-diversity, and reproduction/death rates. It is thus interesting to explore the interpretation that would be provided of the species distribution in Figure 1 by the neutral theory. Under this assumption, in which reproduction and death rates are the same for all species, the spatial distribution of diversity is described by [3], [4], [6], [17]: i) the probability of pairs of conspecific sites (
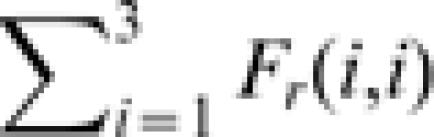
) or bare soil sites (*F_r_(0,0)*) occurring at a distance *r* (hollow squares and circles in Figure 3, respectively); ii) the probability of a vegetated site and a bare soil site being at distance *r* (
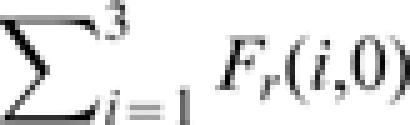
, diamonds in Figure 3); and iii) the probability of heterospecific sites occurring at a distance *r* (e.g. computed as 1−*F_r_(0,0)−F_r_(1,0)−F_r_(1,1),* triangles in Figure 3). We notice here that the number of adjustable parameters is smaller in the neutral model (2 parameters) than in the non-neutral one (6 parameters). However, it is not at all obvious that the non-neutral model should outperform the neutral one, because the number of beta-diversity curves to be fitted is much smaller in the neutral (4 beta-diversity curves) than in the non-neutral case (10 beta-diversity curves). The observed beta-diversity curves computed according to the neutral framework exhibit a correlation which decreases with distance at a rate which is intermediate between those of *Limonium narbonense* and *Sarcocornia fruticosa* and of *Spartina maritima* as obtained from the non-neutral model. Also, any information on a preferential distance between individuals of *Limonium narbonense* and *Sarcocornia fruticosa* is obviously lost, because the two species are not distinguished from each other in a neutral setting. Fitting the observed beta-diversity using the expressions obtained from eq. (1) when *v′_i_ = v′* and *d′_i_ = d′* yields *v′* = *2.2 ·10^−5^* and *d′ = 3.82 ·10^−5^*, corresponding to the modelled beta-diversity curves indicated by solid lines in Figure 3. The parameter values fall between those of *Limonium narbonense* and *Sarcocornia fruticosa* (which were quite similar) and those of *Spartina maritima*, as obtained from the non-neutral model. The shape of the beta-diversity curves and the values of the parameters thus suggest that the neutral assumption amounts to lumping the actual species into a fictitious species with intermediate properties. In particular, the order-of-magnitude differences between non-neutral and neutral values of the parameters suggest that the good agreement between the neutral model and observations in the present case inspires a misleading confidence in the ability of the approach to describe vegetation diversity patterns. The close fit of observed beta-diversity curves by the neutral model is, in fact, obtained by defining a ‘virtual’ species to represent the average reproductive characteristics of actual ones, with a loss of physical significance. These considerations point to the usefulness of a parameter-parsimonious non-neutral model, which is able to capture the heterogeneous properties of individual species and to decipher the competitive strategies embedded in the observed beta-diversity patterns.

## Supporting Information

Methods and Materials S1 Detailed methods and model formulation
(0.17 MB DOC)

Figure S1 Observational soil elevation frequency curves conditional to the presence of the different vegetation species of interest (modified after [14]).
(1.89 MB TIF)
