# Supplementary material for: Non-Neutral Vegetation Dynamics
Source: PLoS One. 2006 Dec 20;1(1):e78. doi: 10.1371/journal.pone.0000078 (PMC1762364; doi:10.1371/journal.pone.0000078)
Supplement: Methods and Materials S1 — Detailed methods and model formulation (0.17 MB DOC) [file pone.0000078.s001.doc]

**Supporting Information**

*Processing and classification of remote sensing data*

The remote sensing data used to map salt-marsh vegetation were acquired by a Multispectral Linescanner sensor on 4 October 2002 by the Falcon II sensor of Toposys GmbH (Ravensburg, Germany). The flight altitude was 500 m, resulting in a 0.5 m ground resolution. The sensor operates in four spectral bands, covering the intervals 450

nm - 490 nm, 500 nm - 580 nm, 580 nm - 660 nm, 770 nm - 890 nm. The data are part of a larger dataset acquired within the EU TIDE research project (www.tideproject.org), which is described in greater detail elsewhere [18].

The application of classification methods for intertidal vegetation mapping requires ground reference data of species presence for classifier training and validation. The dataset used comprises accurately located reference areas, identified concurrently with remote sensing campaigns by use of Differential GPS techniques in the field (accuracy of at least ±1 cm in all three co-ordinate directions), with an extension greater than several times the pixel size. Reference areas were selected to represent each of the species of interest. In order to account for the possible presence of minor quantities of other species within the same area and for the variable density of vegetation, and thus of visible soil, relative ground cover was estimated by acquiring several photographs within each reference area from a digital camera mounted on a 2.5 m high pole (resulting in a resolution of 2 mm). Relative covers were estimated by overlaying a regular grid (with cell size of 25 cm) onto the digital images and by identifying the type of cover characterizing the pixels at the intersections of the grid (at least 96 pixels within every image of about 5.8 m^2^). Reference areas were defined as monospecific, and thus used for classifier training and validation, if covered for more than 60 % by a single species.

The ground truth dataset constructed with this procedure is thus constituted by a set of geocoded reference areas for each of the halophytic species of interest (*Spartina maritima*, *Limonium narbonense*, *Sarcocornia fruticosa*, nomenclature follows Caniglia, G., Contin, G., Fusco, M., Anoè, N. & Zanaboni, A., 1997, Confronto su base vegetazionale tra due barene della laguna di Venezia, *Fitosociologia*, **34**, 111-119.) and for bare soil. Reference areas for the water class were selected directly within the Multispectral Line Scanner scene, in which tidal channels can be easily identified. Several unsupervised and supervised classification procedures were tested on a large dataset including data from satellite and airborne sensors with different geometric and spectral resolutions. The results indicate that classifications from hyperspectral sensors (with up to 100 spectral bands) produce vegetation maps which have a very similar accuracy to those from multispectral sensors with just four bands (visible and near infrared) [18]. The large set of experiments performed with different classifiers and sensors also shows that geometric resolution is crucial for obtaining high accuracy values, because low resolutions (coarser than about 1 m, in the case of the Venice Lagoon) produce highly mixed pixels with an inevitable decrease of the separability of the corresponding spectra. On the basis of these results, the use of the Multispectral Line Scanner data, having a high geometric resolution in the visible and infrared part of the spectrum, is suited to obtain high-accuracy vegetation maps.

It should be noticed that the map in Figure 1 does not include species occurring in minimal amounts (e.g. the vegetation map cannot include species occurring in clusters having a size smaller than the pixel size) and describes the distribution of the dominant vegetation species, which occupy the vast majority of the vegetated area within the study marsh. Estimates based on both field and remote sensing information indicate that less than 3% of the study marsh is occupied by the vegetation species not included in Figure 1. The impact of their presence on the evaluation of the spatial distribution of species diversity is thus deemed negligible.

*Habitat spatial variability*

The model presented allows the description of different competitive strategies by adopting species-dependent birth and death rates, whose spatial dependence is, for simplicity, neglected. As discussed in the main text, the detailed spatial distribution of intertidal vegetation does depend on soil aeration, which locally mainly depends on soil elevation. It is thus important to address the possible impacts of the site topographic variability on the spatial homogeneity assumption on which the model is based. Soil elevation at the San Felice marsh varies between 0.01 m above mean sea level (a.m.s.l.) and 0.68 m a.m.s.l. About 240 differential GPS elevation measurements with ±1 cm vertical accuracy were performed at the site [14]. The soil elevation observations were partitioned on the basis of species presence to produce soil elevation frequency distributions conditioned to the presence of the different vegetation species of interest (Figure S1). The observational frequency distributions indicate that indeed species display preferential elevation ranges and that the mode of each distribution is slightly shifted. However, differences in the frequency distributions are relatively minor in the 10 cm - 40 cm a.m.s.l. interval (in which lies 75 % of San Felice soil elevation values) and justify a spatially-homogeneous zero-order model.

*Model detailed structure*

Let σ**_x_** indicate the state of a generic site **x** among the *N* sites within the 2D model square domain. We adopted periodic boundary conditions for the domain, which result in more straightforward analytical derivations. Note that the specific form of the boundary conditions is not relevant if the characteristic correlation lengths of species distributions are much smaller than the linear size of the system. In this case, in fact, species dynamics are not constrained by the presence of the boundaries (except in their immediate neighborhood). This was found to be the case at our study marsh, as the observational beta-diversity curves indicate that species presence becomes uncorrelated at distances which are much smaller than the size of the whole system (see Figure 2). A site may be either empty (σ**_x_**=0) or occupied by any species *j* among *S* possible species (σ**_x_**=*j*, *j* *∈ {1,…, S}*). Let us denote the generic state of the whole system as $\vec{\sigma}=(\sigma_{1}, \ldots,\sigma_{N})$. The master equation, expressing the probability, $P^{n}\left(\vec{\sigma}\right)()$, that the system be in the generic state $\vec{\sigma}$ at the *n-th* time step may in general be expressed in the following form [24]:

(1)

As discussed in the main text, the model assumes that the state of just a single site is modified at each time step and that the transition probabilities, *W*’s, do not depend on the time step, *n*, in the time interval of interest. We further assume that the transition probabilities for each site only depend on its neighbors’ and its own state so that transition probability matrices take on the following form:

(2)

with:

(3)

where the sum in *z(****x****)* is over the nearest neighbors of ***x***. In this equation, the operator *I* represents the death of a plant or the birth of plants from dormant seeds or from vegetative material transported from other areas of the domain (non-local reproduction mechanism). The operator *J* represents the site colonization by propagule growth from neighboring sites (local reproduction mechanism). In order for the probabilities of mutually-exclusive events to sum to unity it must be:

$\begin{matrix} I_{\sigma,\sigma'}=k\cdot L^{(1)}_{\sigma,\sigma'} \\ J_{\sigma,\sigma'}=\frac{1-k}{q(\mathbf{x})}\cdot L^{(2)}_{\sigma,\sigma'} \\ \sum_{\sigma} L^{(i)}_{\sigma,\sigma'}=1 \end{matrix}$ (4)

where *q(****x****)* is the number of sites neighboring site **x** (four in the present case) and the elements of $L^{(i)}_{\sigma,\sigma'}$ can be interpreted as transition probabilities from state *σ’* to state *σ*. The parameter *k* weighs the probabilities of occurrence of local and non-local reproductions mechanisms (represented by operators *J* and *I* respectively).

We are interested in the probability that two sites, **x** and **y**, host species *i* and *j* at time *n*:

$P_{\mathbf{x},\mathbf{y}}^{n}(i,j)\equiv\left\langle\delta_{i,\sigma(\mathbf{x})}\cdot\delta_{j,\sigma(\mathbf{y})}\right\rangle$ (5)

Because of the homogeneous nature of the model, this function can be written as follows:

$F_{\mathbf{r}}^{n}(i,j)\equiv P_{\mathbf{x},\mathbf{y}}^{n}(i,j)$ (6)

with: ***r****=****x****-* ***y***. An equation describing the time change of *F^n^****_r_****(i,j)* can be derived:

$F_{\mathbf{r}}^{n+1}-F_{\mathbf{r}}^{n}=\frac{1-k}{2}\left[\Delta F_{\mathbf{r}}^{n}+\mathbf{M}F_{\mathbf{r}}^{n}\right]$ (7)

with:

$\mathbf{M}\equiv\frac{2k}{1-k}\left[L^{(1)}\otimes\mathbf{1}+\mathbf{1}\otimes L^{(1)}-2\cdot\mathbf{1}\otimes\mathbf{1}\right]$ (8)

$\Delta F_{\mathbf{r}}^{n}\equiv\sum_{h=1}^{d} \left[F_{\mathbf{r}+\mathbf{e}_{h}}^{n}+F_{\mathbf{r}-\mathbf{e}_{h}}^{n}-2F_{\mathbf{r}}^{n}\right]$ (9)

where **1** is the unit matrix, ⊗ is the tensor product, ***e****_h_* is the vector of unit length along direction *h*, and we have posed that $L^{(2)}_{\sigma,\sigma'}=\delta_{\sigma,\sigma'}$, because plants can only generate conspecific propagules.

We are interested in stationary-state properties, for which $F_{\mathbf{r}}^{n+1}=F_{\mathbf{r}}^{n}=F_{\mathbf{r}}; \Delta F_{\mathbf{r}}^{n}=\Delta F_{\mathbf{r}}$. From eq. (7) it thus follows that:

$\Delta F_{\mathbf{r}}+\mathbf{M}F_{\mathbf{r}}=0$ (10)

The explicit expression of *L^(1)^* is:

$L^{(1)}=\left(\begin{matrix} 1-\sum_{i=1}^{N} v_{i} & d_{1} & \ldots& d_{N} \\ v_{1} & 1-d_{1} & 0 & 0 \\ \vdots& 0 & \ddots& 0 \\ v_{N} & 0 & 0 & 1-d_{N} \end{matrix}\right)$ (11)

where *k d_i_* is the mortality rate for species *i*, and *k⋅ v_i_* is the birth rate due to dormant seeds or transported vegetative material (non-local birth mechanism) for species *i*. Notice that the parameters *k, v_i_* and *d_i_* appear in eq. (10) only within the following combinations:

$v'_{i}=\frac{kv_{i}}{1-k}; d'_{i}=\frac{kd_{i}}{1-k}$ (12)

Also note that the determinant of **M** is equal to zero, so that at least one eigenvalue is equal to zero. Furthermore, **M** is definite non-negative, so that all its eigenvalues are either positive or zero.

Let us introduce the following definitions:

$L^{(1)}\mathbf{t}_{a}^{R}=\lambda_{a}\mathbf{t}_{a}^{R}$ (13)

$\mathbf{t}_{a}^{L}L^{(1)}=\lambda_{a}\mathbf{t}_{a}^{L}$ (14)

$\mathbf{t}_{a}^{L}\cdot\mathbf{t}_{b}^{R}=\delta_{a,b}$ (15)

where *λ_a_* is the eigenvalue of *L^(1)^*, ***t****_a_^L^* and ***t****_a_^R^* are the corresponding left and right eigenvectors.

The solution of Eq.(10) is obtained by left multiplying it by the left eigenvectors of **M**:

$\Delta f_{a,b}(\mathbf{r})+\mu_{a,b} f_{a,b}(\mathbf{r})=0$ (16)

where:

$\mu_{a,b}=\frac{2k}{1-k}(\lambda_{a}+\lambda_{b}-2)$ (17)

are the eigenvalues of **M** and:

$f_{a,b}(\mathbf{r})=\mathbf{v}^{L}_{a,b}\cdot F_{\mathbf{r}}$ (18)

where we defined $\mathbf{v}^{L}_{a,b}\equiv\mathbf{t}^{L}_{a}\otimes\mathbf{t}^{L}_{b}$, while $f_{a,b}(\mathbf{r})$are the projections of $F_{\mathbf{r}}$on the left eigenvectors of **M**.

The solution of the scalar Eq.(16) can be obtained by use of a standard Fourier Transform. The final analytical solution is:

$F_{\mathbf{r}}(i,j)=\sum_{a,b} \left(\mathbf{t}_{a}^{R}\right)_{i}\left(\mathbf{t}_{b}^{R}\right)_{j}\left[\sum_{h} \left(\mathbf{t}_{0}^{R}\right)_{h}\left(\mathbf{t}_{a}^{L}\right)_{h}\left(\mathbf{t}_{b}^{L}\right)_{h}\right]f\left(\mathbf{r},\lambda_{a,b}\right)$ (19)

where:

$\lambda_{a,b}=\frac{k}{1-k}(2-\lambda_{a}-\lambda_{b})$ (20)

$f\left(\mathbf{r},\lambda_{a,b}\right)=\frac{\int_{ 0}^{ \infty} dt e^{-(2+\lambda_{a,b})t}I_{r_{1}}(t)I_{r_{2}}(t)}{\int_{ 0}^{ \infty} dt e^{-(2+\lambda_{a,b})t}I_{0}(t)I_{0}(t)}; \lambda_{a,b}\neq0$ (21)

and e.g. $\left(\mathbf{t}_{a}^{R}\right)_{i}$ is the i-th component of the a-th right eigenvector. *I_z_ (t)* is the modified Bessel function of order *z*, ***r****=(r_1_,r_2_)* and *f(****r****, λ_a,b_=0)=1*.

The expression of the steady-state solution (1):

$F_{\mathbf{r}}(i,j)=\sum_{a,b=0}^{S} c_{i,j,a,b}f_{a,b}\left(r\right)$ (22)

can now be retrieved by posing:

$c_{i,j,a,b}=\left(\mathbf{t}_{a}^{R}\right)_{i}\left(\mathbf{t}_{b}^{R}\right)_{j}\left[\sum_{h} \left(\mathbf{t}_{0}^{R}\right)_{h}\left(\mathbf{t}_{a}^{L}\right)_{h}\left(\mathbf{t}_{b}^{L}\right)_{h}\right]$ (23)

and:

$f_{a,b}\left(r\right)\equiv f\left(\mathbf{r},\lambda_{a,b}\right)$ (24)

Fitting the functions *F_r_(i,j)* in eq. (22) to the observational beta-diversity curves (Figure 2) by the minimization of the mean-square-error yields the *v'_i_* and *d'_i_* values listed in Table 1. We also list the parameter values needed to obtain $c_{i,j,a,b}$ and $\lambda_{a,b}$, namely the eigenvalues $\lambda_{a}$ (Table 2) and the right (Table 3) and left (Table 4) eigenvectors of the matrix *L^(1)^*. The eigenvalues are given in the form *k/(1-k)⋅($\lambda_{a}$-1)*, which is the numerically significant one.

Finally, but quite importantly, the model allows the analytical derivation of the average density, $\rho_{i}=\left(\sum_{x} \left\langle\delta_{i,\sigma_{x}}\right\rangle\right)/N$, for each species *i:*

$\rho_{i}=\sum_{j} \left(kL_{i,j}^{(1)}+(1-k)L_{i,j}^{(2)}\right)\rho_{j}$ (25)

from which the following expressions may be obtained:

$\rho_{0}=\frac{1}{1+\sum_{j=1}^{N} v_{j}/d_{j}}$ (27)

$\rho_{i}=\frac{v_{i}/d_{i}}{1+\sum_{j=1}^{N} v_{j}/d_{j}}; i\neq0$ (28)

*ρ_0_* being the average density of bare soil.

Table 1. Values of model parameters obtained by fitting S. Felice beta-diversity data.

Table 2. Numerical values of *k/(1-k)⋅($\lambda_{a}$-1).*

Table 3. Numerical values of ***t****_a_^R^* .

Table 4. Numerical values of ***t****_a_^L^*.

Figure S1. Observational soil elevation frequency curves conditional to the presence of the different vegetation species of interest (modified after [14]).

| Species | v’ | d’ |
| --- | --- | --- |
| *Spartina maritima* | 1.26⋅10^-4^ | 1.75⋅10^-4^ |
| *Sarcocornia fruticosa* | 3.63⋅10^-6^ | 8.47⋅10^-6^ |
| *Limonium narbonense* | 4.51⋅10^-6^ | 8.78⋅10^-6^ |

**Table 1**

| *a* | 0 | 1 | 2 | 3 |
| --- | --- | --- | --- | --- |
| $\frac{k}{1-k}\left(\lambda_{a}-1\right)$ | 0.00 | -3.04⋅10^-4^ | -1.32⋅10^-5^ | -8.60⋅10^-6^ |

**Table 2**

| *a* | 0 | 1 | 2 | 3 |
| --- | --- | --- | --- | --- |
| ***t****_a_^R^* | $\left(\begin{matrix} 3.75\cdot10^{-1} \\ 2.71\cdot10^{-1} \\ 1.61\cdot10^{-1} \\ 1.93\cdot10^{-1} \end{matrix}\right)$ | $\left(\begin{matrix} -3.77\cdot10^{-1} \\ 3.67\cdot10^{-1} \\ 4.64\cdot10^{-3} \\ 5.76\cdot10^{-3} \end{matrix}\right)$ | $\left(\begin{matrix} 2.93\cdot10^{-1} \\ 2.29\cdot10^{-1} \\ -2.24\cdot10^{-1} \\ -2.98\cdot10^{-1} \end{matrix}\right)$ | $\left(\begin{matrix} 1.41\cdot10^{-2} \\ 1.97\cdot10^{-2} \\ -3.84\cdot10^{-1} \\ 3.60\cdot10^{-1} \end{matrix}\right)$ |

**Table 3**

| *a* | 0 | 1 | 2 | 3 |
| --- | --- | --- | --- | --- |
| ***t****_a_^L^* | $\left(\begin{matrix} 1.00 \\ 1.00 \\ 1.00 \\ 1.00 \end{matrix}\right)$ | $\left(\begin{matrix} -1.15 \\ 1.55 \\ 3.28\cdot10^{-2} \\ 3.40\cdot10^{-2} \end{matrix}\right)$ | $\left(\begin{matrix} 6.54\cdot10^{-1} \\ 7.07\cdot10^{-1} \\ -1.17 \\ -1.29 \end{matrix}\right)$ | $\left(\begin{matrix} 2.36\cdot10^{-2} \\ 2.48\cdot10^{-2} \\ -1.50 \\ 1.17 \end{matrix}\right)$ |

**Table 4**
