# Supplementary figures and images for: Non-Neutral Vegetation Dynamics
Source: PLoS One. 2006 Dec 20;1(1):e78. doi: 10.1371/journal.pone.0000078 (PMC1762364; doi:10.1371/journal.pone.0000078)

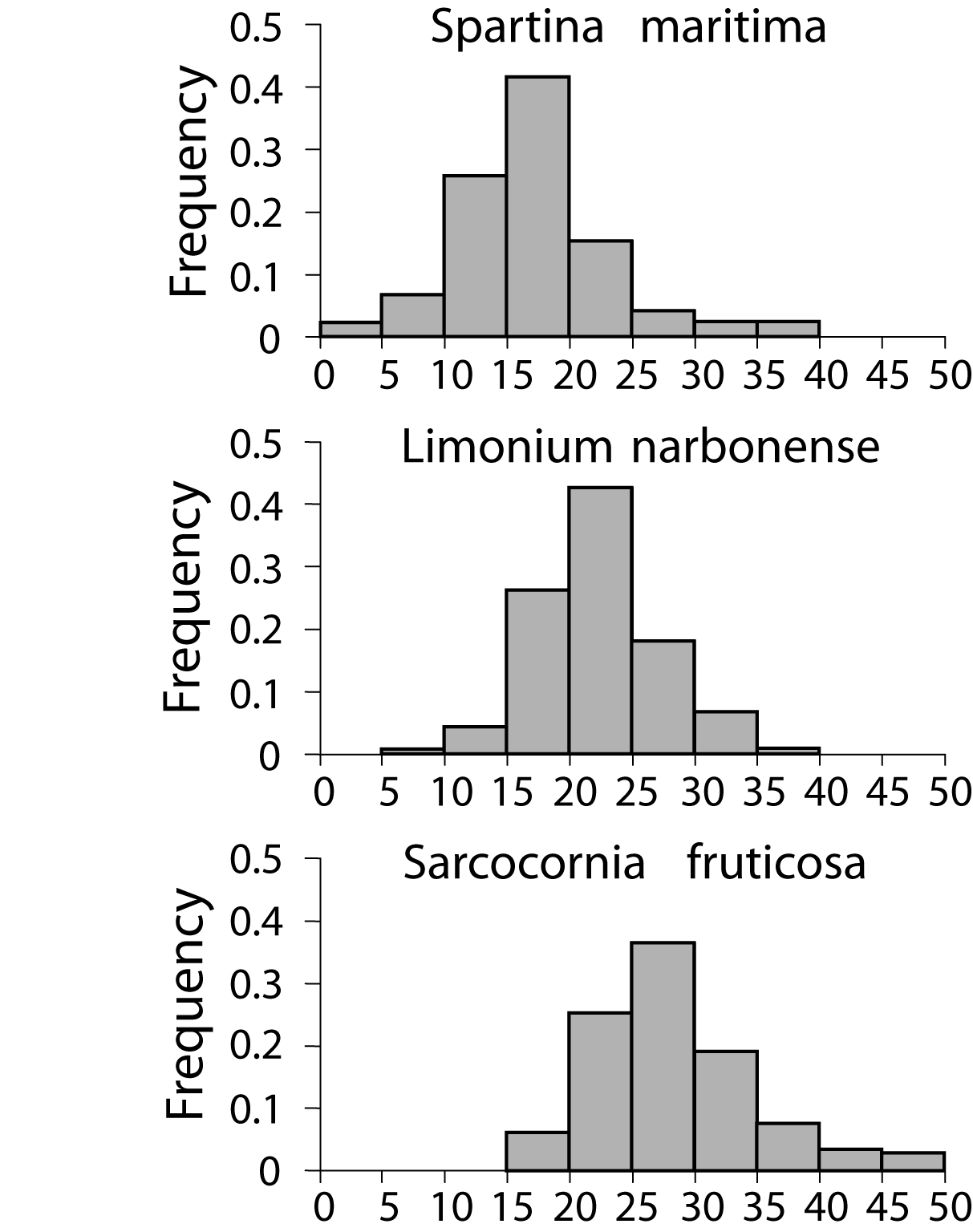

Supplement: Figure S1 — Observational soil elevation frequency curves conditional to the presence of the different vegetation species of interest (modified after [14]). (1.89 MB TIF) [file pone.0000078.s002.tif]
